# Prenatal Diagnosis of Congenital Mesenchymal Hamartoma of Liver: A Case Report

**DOI:** 10.1155/2011/932583

**Published:** 2011-08-24

**Authors:** Sreelakshmi Kodandapani, Muralidhar V. Pai, Vijay Kumar, Kanthilatha V. Pai

**Affiliations:** ^1^Department of OBG, Kasturba Medical College, Manipal University, Manipal 576104, India; ^2^Department of Pediatric Surgery, Kasturba Medical College, Manipal 576104, India; ^3^Department of Pathology, Kasturba Medical College, Manipal 576104, India

## Abstract

Hepatic mesenchymal hamartoma is a rare benign tumor. We present an unusual case of a fetal abdominal cyst, later diagnosed histopathologically to be mesenchymal hamartoma of liver. The organ of origin was indeterminate on both prenatal and postnatal ultrasounds. As there are no specific sonological findings, whenever a large multicystic fetal abdominal cyst is seen, mesenchymal hamartoma should be considered as a possibility.

## 1. Introduction

Increase use of antenatal ultrasound has resulted in greater incidence of prenatal diagnosis of congenital malformations. Many anomalies which manifest in the third trimester are diagnosed, as serial growth of fetus is monitored by ultrasound. Fetal abdominal cyst with normal kidneys and liquor in the third trimester is probably ovarian cyst, mesenteric cyst, or rarely mesenchymal hamartoma of liver as reported in this case. Mesenchymal hamartoma is a cystic benign tumor, but rapid growth to enormous size can lead to fetal hydrops and demise. Case report with management and review of the literature of congenital mesenchymal hamartoma of liver is presented.

## 2. Case Report

**Figure 1:**
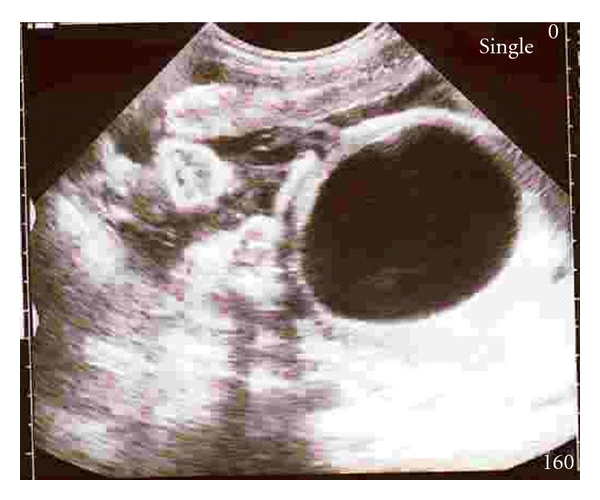
Prenatal ultrasound picture showing intra-abdominal cyst measuring 8 × 8 cm.

A 25-year-old primigravida was referred to us at 38 weeks for management of cardiac disease complicating pregnancy. Incidentally, during antenatal ultrasound, an intra-abdominal anechoic cyst, just above the level of kidneys, measuring 7 × 8 cm was noted (Figure 1). Bladder was visualized separately. Placenta was situated in the upper segment and amniotic fluid index was normal. Intra-abdominal organs, heart, and diaphragm were not displaced. Estimated fetal weight was 2.3 kg suggesting mild IUGR. Provisional diagnosis of either mesenteric, ovarian, or duplication cyst was considered. She had mitral valve prolapse with NYHA class I. Since cyst was neither compressing other organs nor distending abdomen, vaginal delivery was planned. However, cesarean delivery was done for fetal distress. A female baby of 2.15 kg, with APGAR 9 and 10 at 1 and 5 minutes, respectively, was delivered. Postnatal examination of the baby revealed a palpable cystic mass of 10 × 8 cm in the right hypochondrium. No other congenital abnormalities were noted. Baby tolerated feeds well. Ultrasound was suggestive of an anechoic cyst in close relation to the left lobe of liver. Conservative management was advised by pediatric surgeon. Baby was on regular followup. 

**Figure 2:**
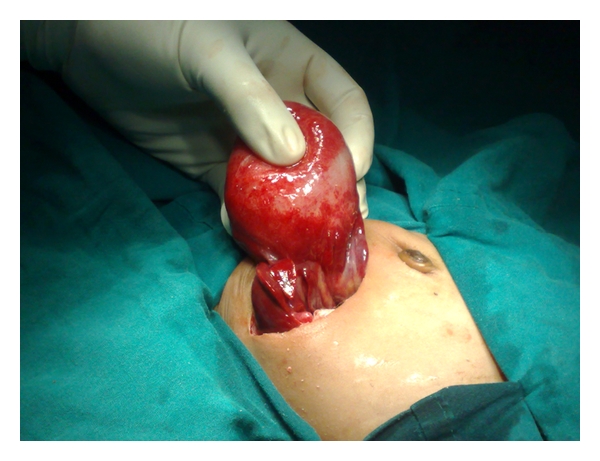
Intraoperative picture showing cyst arising from liver.

**Figure 3:**
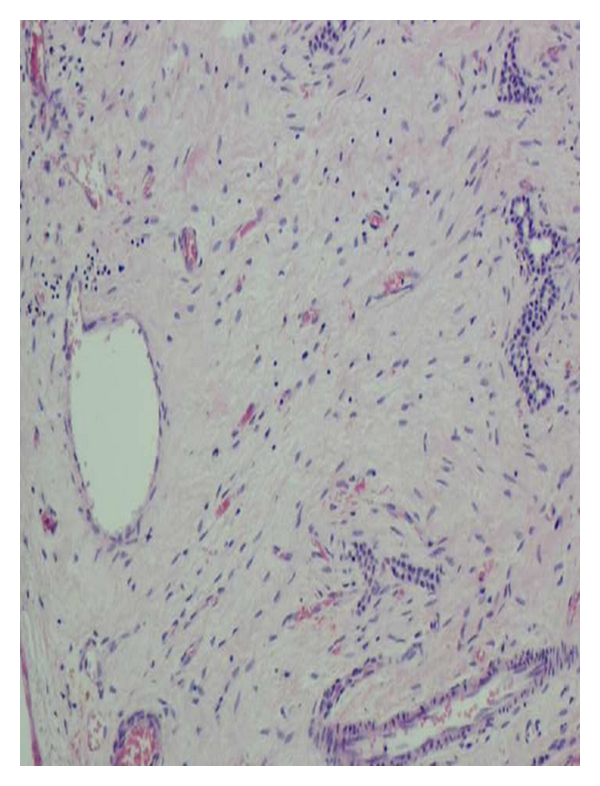
Histopathological image with fluid-filled lakes and devoid of epithelium.

However, laparotomy was performed at 2 months for persistence of cyst. Liver function, renal function tests, and electrolytes were preoperatively normal. At laparotomy, a cystic lesion arising from 5th segment of liver was noted and the same was excised (Figure 2). Histopathology revealed that the cyst was devoid of lining epithelium and was lined by hyalinized fibrous tissue suggestive of mesenchymal hamartoma of liver (Figure 3).

Postoperative period was uneventful. After 2 months of surgery, a small cystic lesion (4 × 1 cm) was seen arising from the inferior part of right lobe of liver. It was decided to followup, the cyst. At the end of 2 months of postoperative follow up the cystic lesion reduced in size to 5 mm and baby was asymptomatic with good catch up growth. 

## 3. Discussion

Hepatic tumors are rare and comprise of 5% of the total neoplasms in fetal and neonatal period [7]. After hemangiomas, mesenchymal hamartomas are the second most common hepatic tumours in childhood [8]. Hepatic mesenchymal hamartoma is a benign tumor, defined as an excessive focal overgrowth of mature normal cells and stroma native to the liver. Hamartomas are devoid of lining epithelium with hemorrhage and necrosis. They are well circumscribed and are away from the biliary ducts. Liver architecture and function is well maintained. With the advent of high resolution ultrasound, these can be detected prenatally as intra-abdominal cysts of unknown origin. Typically, hamartomas of liver are not associated with any anomalies, but associations with congenital heart disease, gut malrotation, omphalocele, myelomeningocele, and biliary atresia have been reported [9]. Prognostic factors are period of gestation of presentation, tumor size, rate of growth, and associated anomalies.

In the largest series by Isaacs Jr, 45 cases of mesenchymal tumors are reported over a period of 35 years (1970–2005) [10]. A total of 14 cases were antenatally diagnosed in this series and most common presentation was abdominal cyst with a mean gestational age of 35 weeks (15–40). While 64% underwent surgical resection, only 10 untreated survived. Most common cause of death was rapidly progressive tumor with respiratory distress. Overall survival was 64% in this series [10]. Slow growth and late onset presentation of cyst in our case was a favorable factor.

Early onset of presentation, rapidly progressing tumor, and polyhydramnios compression of arteries are poor prognostic factors which were seen in cases reported by Dickinson et al., Tsao et al., and Laberge et al. [4–6] (Table 1). Thus, these patients should be on regular followup. Rapidly growing tumor may require antenatal aspiration of cyst as was performed in one of the cases reported by Tsao et al. [5]. 

Cesarean delivery is not per se indicated unless it is a big tumor anticipating abdominal dystocia as can be inferred from Table 1. Recurrence and malignant transformation is rarely observed, hence, warrants careful follow up [11]. Our case has been followedup for 6 months at the time of reporting and has appropriate growth and neurodevelopment.

## Figures and Tables

**Table 1:** Congenital mesenchymal hamartoma of liver: presentation and management.

Author, year, number of cases (*n*)	Gestational age (weeks)	Presentation/mode of delivery	Management	Post-op course followup	Tumour size
Kamata et al. [1] 2003 *n* = 1	30	Rapidly growing cyst with maternal hypertension, anemia, preterm labor. Cesarean delivery	Surgical resection	3-year followup: alive and healthy	7 × 6 × 5 cm
Bartho et al. [2] 1992 *n* = 1	31	Abdominal cyst. Cesarean delivery	Hepato lobectomy	NA	7 × 4 cm
Tovbin et al. [3] 1997 *n* = 1	29	Abdominal cyst. Vaginal delivery	Surgical excision	15th postnatal day	10 × 8 cm
Dickinson et al. [4] 1999 *n* = 1	26	Progressing abdominal mass fetal hydrops, fetal demise. Vaginal delivery	Nil	Nil	8 × 7 × 6 cm
Tsao et al. [5] 2002 *n* = 2	Fetus 1: 35	Fetus 1: rapidly growing mass, vaginal delivery. Fetus 2: rapidly progressing mass, fetal hydrops	Fetus 1: antenatalaspiration postnatal laparoscopic excision of cyst. Fetus 2: excision, neonatal death	Fetus 1: two-week postoperative period was normal Fetus 2: autopsy showed hamartoma umbilical vein compression	Fetus 1: 9 × 6 cm Fetus 2: 13 × 13 × 7 cm weighed 635 g
Laberge et al. [6] 2005 *n* = 1	23	Abdominal cyst polyhydramnios, fetal hydrops, fetal demise	Nil	Placental villous hyperplasia	

NA: not available.
